# New chlorogenic acid derivatives and triterpenoids from Scorzonera aucheriana

**DOI:** 10.3906/kim-2009-17

**Published:** 2021-02-17

**Authors:** İshak ERİK, Kamil COŞKUNÇELEBİ, Serdar MAKBUL, Nurettin YAYLI

**Affiliations:** 1 Department of Pharmacognosy, Faculty of Pharmacy, Karadeniz Technical University, Trabzon Turkey; 2 Department of Biology, Faculty of Science, Karadeniz Technical University, Trabzon Turkey; 3 Department of Biology, Faculty of Science and Arts, Recep Tayyip Erdoğan University, Rize Turkey

**Keywords:** *Scorzonera aucheriana*, chlorogenic acid analogs, triterpenes

## Abstract

Chromatographic separation of*n*-hexane and ethyl acetate fraction of a crude methanol extract obtained from aerial parts of the*Scorzonera aucheriana*DC collected from Sivas province of Turkey yielded nine natural compounds; two new 3-caffeoyl-quinic acid analogs (**1**-**2**), one new taraxasterol oleate (**3**), and six known triterpenoids taraxasterol (**4**), taraxasterol acetate (**5**), ptiloepoxide (**6**), lupeol (**7**), lupeol acetate (**8**), and*β*-sitosterol (**9**) were characterized. The structures of the isolated compounds were elucidated on the basis of NMR (^1^H,^13^C, COSY, HMBC, HSQC, and TOCSY), UV, FT-IR and LC-Q-TOF-MS spectrometric data.

## 1. Introduction

Treatment with plants is as old as human history. Mankind has benefited from natural herbal resources to protect health and treat against diseases. The isolation and characterization of the active compounds found in plants started to develop from the beginning of the 19th century with developing technology. Numerous natural compounds in the scientific process, compounds such as morphine, penicillin, codeine, atropine, resveratrol, and digoxin, were isolated from natural sources and took place in the treatment processes. The isolation of the compounds from natural sources and the clarification of their structures are extremely important in the scientific process as they have the potential to become a new drug-active substance. Due to the potential of natural compounds to be exemplary molecules in active substance synthesis, studies in this field are considered to be inevitable for serving humanity. Scorzonera L. species are known to be used in complementary medicine and consumed as a vegetable in the world and Turkey [1-4].

The genus Scorzonera L. is a member of tribe Cichorieae (Asteraceae), and it is represented by around 180-190 species in the worldwide [1-2]. The major center in terms of diversity is the arid and mountainous Irano-Turanian region [3]. The genus is represented by perennial herbs often having a caudex or tuber under ground [2]. There are 52 species in Turkey and 31 of them are endemic [4]. Some members of this genus are consumed as a vegetable in Turkey [5-6].*S. aucheriana*known as “Buz Tekesakalı” in Turkey is caulescent, herbaceous perennial herbs endemic to Turkey [4]. Their rootstock is thick and crowned by decayed leaf remains [2]. Members of Scorzonera including*S. aucheriana*are characterized by a rich latex ingredient [2].

It has been stated that different Scorzonera species are used as pain relievers among the public, and scientific studies in our country have analgesic, antiinflammatory and wound-healing effects [7-11].*Scorzonera mongolica*Maxim. and*Scorzonera austriaca*Willd. are grown in China and are used in the treatment of fever reduction, boils, and nipple inflammation [12-14].*Scorzonera divaricata*Lipsch root and aboveground parts are used in the treatment of ulcers and malignant stomach tumors;*Scorzonera pseudodivaricata*Turcz is used in febrile conditions caused by pulmonary edema, diuretic, and viral infection [15].*Scorzonera cretica*Willd., Greek cuisine is used in dishes such as savory [16].*S. pseudodivaricata*and*S. divaricata*species grow in Mongolia and are used for medical purposes.*Scorzonera hispanica*is the most commonly grown and cultivated ornamental plant in Europe, as well as being used to treat colds, lung diseases, appetizing, mucolytic, stomatic, diuretic, antipyretic effects, snake bites, and it is also added to coffee [17].*Scorzonera humilis*L. is used in wound healing and gastrointestinal disorders [18]. Rootstock and fresh shoots of some of the Scorzonera member grown in Turkey are consumed fresh or cooked under the name of ‘manger’. It has been stated that these plants are used for the purpose of removing high blood pressure, kidney diseases, diabetes, moxibustion, infertility, and vascular stiffness [5,19].

Phytochemical studies of Scorzonera species gave the guaianolid class sesquiterpene lactones, flavonoids (such as apigenin, kaempferol, luteolin and quercetin), cafeoyl quinic acids (chlorogenic acid), coumarins, lignans, neolignans, bibenzyl derivates, benzyl phthalides, stilbenoids and triterpenoid class of natural compounds. Studies show that the Scorzonera species is quite rich in terpenic and phenolic compounds [15,20].

Bioactivity studies have also been conducted on Scorzonera genus; the dihydrostilben derivative, Skorzodihidrostilben A, B, C, D, and E compounds obtained from the above ground parts of*Scorzonera radiata*have reported to have very strong antioxidant activity [21]. Antioxidant (antiproliferative effect on L5178Y mouse lymphoma cells) was investigated in bioactivity directed isolation studies of methanolic extracts prepared from above ground parts of*S. pseudodivaricata*and*S. divaricata*plants. Feruloyl podospermic acid A and B compounds isolated from*S. divaricata*have been investigated; their DPPH radical scavenging effect has been searched and reported to have stronger antioxidant activity standard [15].

The immunomodulatory effect of syringarezinol glycoside I isolated from*S. hispanica*was determined [22]. The antinociceptive activities of Scorzonera species have been tested and it has been reported that*Scorzonera latifolia*(Fisch. & Mey.) DC. is the most effective and those antinociceptive effective taraxasterol acetate and taraxasterol myristate compounds are isolated by bioactivity directed fraction [10]. It was reported that the pilatifillozide compound was isolated from the cytotoxic activated directed study of*S. pseudodivaricata*plant [15]. The cytotoxic activity of thyrolobibenzyls obtained from*S. humilis*, antibacterial activity against P-388 cells, and antifungal activity against*Candida albicans*and*Trichophyton mentagrophytes*were investigated, but it was reported that the compounds showed no activity [18,23]. Flavonoids constitute an important class of natural compounds with polyphenolic structure. Flavonoid compounds are known to have antioxidant activity associated with diseases such as Alzheimer’s, cancer, and atherosclerosis. Phenolic compounds have antiinflammatory, anticarcinogenic, antimutagenic, and antioxidative activities due to their capacity to modulate cellular enzyme functions. Polyphenolic compounds are widely used in complementary therapy in nutraceutical, pharmaceutical, medical, and cosmetic applications [24].

According to the best of our knowledge, there is no any secondary metabolite isolation, structure determination, and biological activity studies with*S. aucheriana*, which we plan to work on. Chemical diversity of Scorzonera species is of great importance in terms of the inclusion of new therapeutic compounds in the scientific world. In addition, the determination of the compounds responsible for the biological activity indicates that the study within this plan will be of special importance.

## 2. Materials and methods

### 2.1. Plant materials

*S. aucheriana*was collected in July 2018 from Sivas province, Zara district, Yarağıl region in Turkey. The plant was identified by Prof. Kamil Çoşkunçelebi by using Flora of Turkey [2] and taxonomical conspectus of Turkish Scorzonera [4]. Voucher (Makbul 244 & Coşkunçelebi) was deposited in the Herbarium of Biology (KTUB) at Karadeniz Technical University, Turkey. The plant cleaned to remove impurities, dried, and stored in air-tight container before use.

### 2.2. Chromatographic and instrument methods

Optical rotations were measured on an Automatic AA-5 series polarimetry. UV spectra were obtained with a Spectrostar nano BMG labtech spectrometer. Infrared spectra were obtained with a PerkinElmer 1600 FT-IR (ATR) (4000-400 cm^-1^) spectrometer (PerkinElmer, Inc., Waltham, MA USA). The mass spectral analyses were carried out on Agilent 6230A LC-Q-TOF-MS. Shimadzu QP2010 ultra GC-FID/MS was used to identify the FAMEs. Melting points were determined using Thermo-var apparatus fitted with a microscope and uncorrected. ^1^H and ^13^C NMR, along with 2D NMR spectra, were obtained on a Bruker 400 MHz NMR spectrometer (400 MHz for ^1^H, 100 MHz for ^13^C), using TMS as an internal standard. CD_3_OD and CDCl_3_ were used as NMR solvent. ACD NMR program was used for the elucidation of isolated compounds. Chemical shifts were expressed in δ (ppm) and coupling constants (J) were reported in hertz (Hz). CC was carried out on silica gel (Kieselgel 60, 320-400 mesh), RP-18 silica gel, and PTLC (Silica gel HF_254_, 20 × 20 cm, 0.5 mm). TLCs were carried on silica gel (Kieselgel 60 F_254_, Merck) plates, and the spots were visualized by UV lamp or spraying with 20% H2SO4 and heating.

### 2.3. Extraction and isolation

Dried powdered aerial parts of*S. aucheriana*(833 g) were macerated three times with methanol (4 L) for 72 h at room temperature on a stirring. Total extracts were evaporated under vacuum at a temperature not exceeding 40 ºC using a chiller (-10 oC) to yield crude extracts (60.6 g). The crude methanol extract (42.96 g) was dissolved in MeOH-H_2_O (2:8) and then successively fractionated with n-hexane (50 mL × 3), chloroform (50 mL × 3), ethyl acetate (50 mL × 3) and water (80 mL) to yield 12.9 g, 1.8 g, 4.6 g, and 20.9 g, respectively.

The crude ethyl acetate fraction (4.6 g) was subjected to CC (Kieselgel 60, 320-400 mesh) using increased polarity of*n*-hexane-AcOEt (98:2, 97:3, 95:5, 93:7, 90:10, 85:15, 80:20, 75:25, 70:30, 65:35, 60:40 ,55:45, 50:50, 40:60, 30:70, 20:80, 10:90, 0:100 ml, each), and AcOEt-methanol (98:2, 97:3, 95:5, 93:7, 90:10, 85:15, 80:20, 75:25, 70:30, 65:35, 60:40 ,55:45, 50:50, 40:60, 30:70, 20:80, 10:90, and 0:100) solvent mixture to give 106 fractions (~35 mL, each, EA). After the TLC control, some of the fractions were combined and EA-Fr 83-91 (135 mg) was further separated by PTLC (silica gel HF_254_, 20×20, 0.5 mm, 2 plates) using AcOEt-MeOH-H_2_O (70:30:4.5) mobile phase to provide A (16 mg), B (15.4 mg), C (20.9 mg), and D (58.5 mg) bands. NMR data of bands A-C seemed to be so complex mixture and not further study was done. Band D was further separated by reverse phase CC (RP-18, silica gel) using decreased polarity of H_2_O:MeOH (100:0, 95:5, 90:10, 85:15, 80:20, 70:30, 60:40, 50:50, 40:60, 30:70, 20:80, 10:90, and 0:100 ml, each) mobile phase to give 45 subfraction (30 ml, each). After the TLC control, EA-SFr 12 and EA-SFr 18 afforded compounds 1 (6.4 mg) and 2 (6.2 mg), respectively.

The crude*n*-hexane fraction (12.9 g) was subjected to CC (Kieselgel 60, 320-400 mesh) using increased polarity of*n*-hexane-chloroform (100:0, 90:10, 80:20, 70:30, 60:40, 50:50, 40:60, 30:70, 20:80, 10:90, and 0:100 ml each), chloroform- AcOEt (100:0, 98:2, 95:5, 90:10, 80:20, 70:30, 60:40, 50:50, 40:60, 20:80, and 0:100 ml each), and ethyl acetate-metanol (100:0, 50:50, and 0:100 ml each) mobile phase to give 63 fractions (H) (~40 ml each). After the TLC control, some of the fractions were combined and H-Fr 34-42 (610 mg) was purified by repeated CC (Kieselgel 60, 320-400 mesh) with increasing polarity of*n*-hexane-CHCl_3_ (100:0, 99:1, 98:2, 96:4, 92:8, 88:12, 85:15, 80:20, 75:25, 65:35, 60:40, 50:50, 40:60, 35:65, 30:70, 25:75, 20:80, 15:85, 10:90, 5:95, and 0:100 mL, each), and*n*-hexane-AcOEt solvent mixture (100:0, 95:5, 90:10, 85:15, 80:20, 70:30, 60:40, 50:50, 30:70, 10:90, and 0:100 ml, each) to give 65 subfractions (SFr) (~15 ml, each). After the TLC control, H-SFr 7 and H-SFr 13 gave the compounds 6 (12.2 mg) and 4 (25.8 mg), respectively.

H-Fr 15-17 (2.24 g) was purified by again CC (Kieselgel 60, 320-400 mesh) increasing polarity of*n*-hexane-AcOEt (100:0, 99:1, 98:2, 97:3, 96:4, 95:5, 64:6, 63:7, 92:8, 91:9, 90:10, 89:11, 88:12, 87:13, 86:14, 85:15, 84:16, 83:17, 82:8, 81:19, 80:20, 76:24, 70:30, 50:50, 0:100 ml, each) to give 50 subfractions (~20 mL, each). After the TLC control, H-SFr 19 and H-SFr 34 afforded compounds 7 (8.1 mg) and 9 (9.8 mg), respectively. After the TLC control, crude n-hexane fractions (H-Fr) 10-14 were combined and purified by CC (Kieselgel 60, 320-400 mesh) with increasing polarity of n-hexane-CHCl_3_ (100:0, 95:5, 90:10, 80:20, 70:30, 60:40, 55:45, 50:50, 45:55, 40:60, 30:70, 20:80, 0:100 mL, each), and CHCl_3_-AcOEt (100:0, 95:5, 90:10, 80:20, 60:40, 40:60, 20:80, 0:100 mL, each) to give 78 subfraction (H-SFr) (~15 ml, each). After the TLC control, H-SFr 27 afforded compound 3 (30.2 mg). Subfractions (H-SFr1) 35 and 36 combined (100.3 mg) and were further purified by CC (Kieselgel 60, 320-400 mesh) with increasing polarity of petroleum ether-dichloromethane (100:0, 99:1, 98:2, 97:3, 96:4, 95:5, 94:6, 93:7,92:8, 91:9, 90:10, 89:11, 88:12, 83:17, 84:16, 85:15, 84:16, 83:17, 82:18, 81:19, 80:20, 75:25, 70:30, 60:40, 50:50, 40:60, 30:70, 20:80, 0:100 ml, each) to give 66 subfraction-2 (H-SFr2) (~15 ml, each). H-SFr2 16-18 and SFr2 19-21 afforded compounds 8 (4.7 mg) and 5 (4.9 mg), respectively.

**Compound 1 (Methyl 1-(2-methylcyclopropyl-1-carbonyloxy) chlorogenate)**

Colorless semisolid, m.p. 198-200 °C; [α]D24 +90.9 (c 0.00015, Methanol); Rf: 0.45 (AcOEt-methanol-water, 70:27:3); UV (Methanol) λmax (nm): 216, 245, 324. FT-IR (ATR, cm^-1^): 3368 (-OH), 3050 (=CH), 2949, 2860 (-CH), 1658, 1594 (C=O), 1490, 1450 (C=C) 1268, 1121, 1033 (C-O); C22H26O10, LC-QTOF-MS: m/z (%) [M+Na]^+^ 473.1412 (08), calc. 473.1417; [M-OCH_3_]^+^ 419.1189 (08), calc. 419.1186; [M-CH_3_COO-CH_3_-H]^+^ 375.0661 (30), calc. 375.0663; [M-99+CO_2_+2Na]^+^ 353.0841 (100), calc. 353.0838. ^1^H and ^13^C NMR data see table 1.

**Table 1:** NMR data of compound 1 (CD_3_OD, 400 MHz NMR).

No	C (APT)	^1^H (COSY)	^13^C (HSQC)
1	C	-	76.37
2	CH_2_	2.15 (m, ^1^H, H-2a)2.05 (m, ^1^H, H-2b)	37.64
3	CH	5.39 (m, ^1^H,)	71.72
4	CH	3.70 (d, J = 8.3 Hz, ^1^H)	73.77
5	CH	4.14 (bs, ^1^H)	71.05
6	CH_2_	2.20 (m, ^1^H, H-6a)1.97 (m, ^1^H, H-6b)	39.31
7	C	-	169.01
	OCH_3_	3.33 (s, 3H)	48.45
1′	C	-	124.95
2′	CH	7.05 (d, J = 1.8 Hz, ^1^H)	112.76
3′	C	-	146.19
4′	C	-	146.65
5′	CH	6.73 (d, J = 8.1 Hz, ^1^H)	115.25
6′	CH	6.92 (d, J = 8.1 Hz, ^1^H)	121.95
7′	CH	7.58 (d, J = 16.0 Hz, ^1^H)	151.01
8′	CH	6.26 (d, J = 16.0 Hz, ^1^H)	115.25
9′	C	-	168.25
1′′	C	-	179.78
2′′	CH	1.05-0.88 (m, ^1^H)	20.98
3′′	CH	1.05-0.88 (m, ^1^H)	22.18
4′′	CH_2_	1.78 (m, ^1^H)1.60 (m, ^1^H)	24.21
5′′	CH_3_	1.02 (d, J = 6.8 Hz)	10.90

**Compound 2 (3,4-Bis[(3**′**,4-dioxo-1**′**,3**′**,5**′**,6**′**-tetrahydrospiro[cyclohexa-2,5-diene-1,4**′**-cyclopenta[c]-furan]-1**′**-yl)] chlorogenic acid)**

Colorless semisolid, m.p. 220-224 °C; [α]_D_^24^ +133 (c 0.00033, Methanol); R_f_: 0.70 (AcOEt-metanol-water, 70:27:3); UV (Methanol) λmax (nm): 218, 243, 327; FT-IR (ATR, cm^-1^): 3421 (-OH), 3065 (=CH), 2946, 2851 (-CH), 1703, 1657, 1597 (C=O), 1490, 1417 (C=C), 1270, 1033 (C-O); C_40_H_34_O_15_, LC-QTOF-MS: m/z (%) [M-H]^+^ 753.6709 (10), calc. 753.6704; [M-H_2_O-H]^+^ 735.6552 (05), calc. 735.6558; [M-CO_2_-2H_2_O+H]^+^ 675.6651 (100), calc. 675.6669. ^1^H and ^13^C NMR data see table 2.

**Table 2:** NMR data of compound 2 (CD_3_OD, 400 MHz NMR).

No	C (APT)	^1^H (COSY)	^13^C (HSQC)	HMBC
1	C	-	76.38	
2	CH_2_	2.16 (m, ^1^H, H-2a)1.97 (m, ^1^H, H-2a)	37.62	
3	CH	5.40 (m, ^1^H)	71.71	
4	CH	3.71 (d, J = 4.0 Hz, ^1^H, H-4)	73.68	
5	CH	4.13 (bs, ^1^H, H-5)	71.18	
6	CH_2_	2.12 (m, ^1^H, H-6a)2.02 (m, ^1^H, H-6b)	39.29	
7	C	-	174.20	
1′	C	-	122.21	
2′	CH	7.05 (d, J = 1.8 Hz, ^1^H)	112.81	C3′, C1′
3′	C	-	152.01	
4′	C	-	145.73	
5′	CH	6.77 (d, J = 7.8 Hz, ^1^H)	113.26	
6′	CH	6.95 (d, J = 7.8 Hz, ^1^H)	121.72	
7′	CH	7.59 (d, J = 16.0 Hz, ^1^H)	146.02	C9′, C6′, C2′
8′	CH	6.30 (d, J = 16.0 Hz, ^1^H)	115.17	C1′
9′	C	-	168.95	
1′′	CH	7.00 (s, 2H)	105.80	C4′, C5′, C6′, C3′′, C6a′′
3′′	C	-	163.67	
3a′′	C	-	149.33	
4′′	C	-	52.13	
5′′	CH_2_	3.57 (t, J = 7.3 Hz, 4H)	32.93	C4′′, C6′′, C3a′′
6′′	CH_2_	2.25 (t, J = 7.3 Hz, 4H)	37.63	C4′′, C5′′, C4′′′, C2′′′, C3a′′
6a′′	C	-	167.93	
1′′′	C	-	52.13	
2′′′, 6′′′	CH	7.11 (d, J = 12.0 Hz, 4H)	155.34	C4′′, C2′′′, C5′′, C4′′′
3′′′, 5′′′	CH	6.25 (d, J = 12.0 Hz, 4H)	126.03	C4′′
4′′′	C	-	187.83	

**Taraxasterol oleate (3)**

Colorless semisolid, m.p. 47-48 °C; [α]_D_^24^ +175.4 (c 0.0017, Methanol); Rf: 0.68 (*n*-hexane-AcOEt, 9.5:0.5); FT-IR (ATR, cm^-1^): 3055 (=CH), 2935, 2890 (-CH), 1657 (C=O), 1495, 1440 (C=C), 1280 (C-O); C_48_H_82_O_2_, LC-QTOF-MS: m/z (%) [M]^+^ 691.1509 (6), calc. 691.1512; [M-C_4_H_9_-H]^+^ 633.0808 (12), calc. 633.0818; [M-oleic acid+H]^+^ 409.7036 (10), calc. 409.7032; [Aglycone+CO_2_-H]^+^ 468.7038 (45), calc. 409.7031.

**Aglycone**: ^13^C NMR (100 MHz, CDCl_3_) δ (ppm): 38.4 (C-1), 23.7 (C-2) , 80.6 (C-3), 37.8 (C-4), 55.4 (C-5), 18.1 (C-6), 34.0 (C-7), 40.9 (C-8), 50.3 (C-9), 37.0 (C-10), 21.4 (C-11), 26.1 (C-12), 39.1 (C-13), 42.0 (C-14), 26.6 (C-15), 38.3 (C-16), 34.5 (C-17), 48.6 (C-18), 39.3 (C-19), 154.6 (C-20), 25.6 (C-21), 38.8 (C-22), 27.9 (C-23), 16.3 (C-24), 16.6 (C-25), 15.8 (C-26), 14.7 (C-27), 19.5 (C-28), 25.5 (C-29), and 107.2 (C-30); ^1^H NMR (400 MHz, CDCl_3_) δ (ppm): 4.42 (m, ^1^H, H-3), 0.78 (s, 3H, H-23), 0.77 (s, 3H, H-24), 0.81 (s, 3H, H-25), 0.81 (s, 3H, H-26), 0.83 (s, 3H, H-27), 0.86 (s, 3H, H-28), 0.89 (d, J=6.8 Hz, 3H, H-29), 4.44 (brs, H-30a), 4.40 (brs, H-30b)

**Glycone**:^13^C NMR (100 MHz, CDCl_3_) δ (ppm): 173.1 (C=O), 130.1 (=CH), 129.6 (=CH), 34.87 (CH_2_), 31.95 (CH_2_), 29.73 CH_2_), 29.70 (CH_2_), 29.67 (CH_2_), 29.61(CH_2_), 29.49 (CH_2_), 29.39 (CH_2_), 29.29 (CH_2_), 29.20 (CH_2_), 29.20 (CH_2_), 27.21(CH_2_), 25.19 (CH_2_), 22.72 (CH_2_), 14.1 (CH_3_); ^1^H NMR (400 MHz, CDCl_3_) δ (ppm): 5.32 (m, 2H, H-9, H-10), 2.33 (t, J = 6.8 Hz, 2H, H-2), 1.20 (t, J = 6.8 Hz, 3H).

**Taraxasterol (4)**

R_f_ : 0.42 (Petroleum ether-AcOEt, 9:1); ^13^C NMR (100 MHz, CDCl_3_) δ (ppm): 154.7 (C-20), 107.2 (C-30), 79.0 (C-3), 28.0 (C-23), 15.4 (C-24), 16.3 (C-25), 15.9 (C-26), 15.4 (C-7), 19.5 (C-28), and 25.6 (C-29); ^1^H NMR (400 MHz, CDCl_3_) δ (ppm): 4.60 (brs, H-30a), 4.62 (brs, H-30b), 3.21 (d, J=6.3,13.2, H-3), 0.85 (s, 3H, H-23), 0.87 (s, 3H, H-24), 1.01 (s, 3H, H-25), 0.93 (s, 3H, H-26), 0.85(s, 3H, H-27), 0.77 (s, 3H, H-28), and 1.02 (d, 3H, H-29).

**Taraxasterol acetate (5)**

R_f_: 0.39 (Petroleum ether-dichloromethane, 7:3); ^13^C NMR (100 MHz, CDCl_3_) δ (ppm): 171.0 (OC=O), 154.70 (C-20), 107.1 (C-30), 80.99 (C-3), 27.8 (C-23), 16.5 (C-24), 15.8 (C-25), 16.3 (C-26), 14.6 (C-27), 19.4 (C-28), 25.5 (C-29), and 21.3 (CH_3_CO); ^1^H NMR (400 MHz, CDCl_3_) δ (ppm): 4.62 (brs, ^1^H, H-30a), 4.60 (brs, ^1^H, H-30b), 4.45 (m, ^1^H, H-3), 2.05 (s, 3H, CH_3_CO), 0.85 (s, 3H, H-23), 0.87 (s, 3H, H-24), 1.01 (s, 3H, H-25), 0.93 (s, 3H, H-26), 0.85 (s, 3H, H-27), 0.77 (s, 3H, H-28), and 1.02 (d, 3H, H-29).

**Ptiloepoxide (6)**

R_f_: 0.32 (*n*-Hexane-AcOEt, 8:2); ^13^C NMR (100 MHz, CDCl_3_) δ (ppm): 151.3 (C-20), 112.0 (C-30), 78.9 (C-3), 64.0 (C-22), 56.1 (C-21), 28.0 (C-23), 15.4 (C-24), 16.2 (C-25), 15.9 (C-26), 14. 8 (C-27), 15.1 (C-28), and 27.2 (C-29); ^1^H NMR (400 MHz, CDCl_3_) δ (ppm): 5.06 (s, ^1^H, H-30a), 4.87 (s, ^1^H, H-30b), 3.47 (d, J = 4.6 Hz, ^1^H, H-21), 3.21 (dd, J = 11.3, 5.0 Hz, ^1^H, H-3), 2.91 (d, J = 4.6 Hz, ^1^H, H-22), 0.97 (s, 3H, H-23), 0.77 (s, 3H, H-24), 0.84 (s, 3H, H-25), 1.02 (s, 3H, H-26), 0.95 (s, 3H, H-27), 0.81 (s, 3H, H-28), and 1.05 (d, J = 6.8 Hz, 3H, H-29).

**Lupeol (7)**

R_f_: 0.35 (Petroleum ether-AcOEt, 9:1); ^13^C NMR (100 MHz, CDCl_3_) δ (ppm): 151.0 (C-20), 109.3 (C-29), 79.0 (C-3), 28.0 (C-23), 15.4 (C-24), 16.1 (C-25), 16.0 (C-26), 14.6 (C-27), 18.0 (C-28), and 19.3 (C-30); ^1^H NMR (400 MHz, CDCl_3_) δ (ppm): 4.69 (bs, J = 0.3 Hz, ^1^H, H-29a), 4.57 (bs, J = 0.3 Hz, ^1^H, H-29b), 3.20 (dd, J = 10.4, 4.9 Hz, ^1^H, H-3), 1.00 (s, H-23), 0.76 (s, H-24), 0.83 (s, H-25), 1.03 (s, H-26), 0.97 (s, H-27), 0.79 (s, H-28), and 1.68 (s, H-30).

**Lupeol acetate (8)**

R_f_: 0.33 (Petroleum ether-dichloromethane, 7:3); ^13^C NMR (100 MHz, CDCl_3_) δ (ppm): 171.09 (OC=O), 151.0 (C-20), 109.4 (C-29), 80.99 (C-3), 27.6 (C-23), 16.7 (C-24), 16.4 (C-25), 16.2 (C-26), 14.7 (C-27), 18.1 (C-28), 19.6 (C-30), and 21.3 (CH_3_CO); ^1^H NMR (400 MHz, CDCl_3_) δ (ppm): 4.69 (bs, J = 0.3 Hz, ^1^H, H-29a), 4.57 (bs, J = 0.3 Hz, ^1^H, H-29b), 4.48 (m, ^1^H, H-3), 2.05 (s, CH_3_CO), 0.86 (s, H-23), 0.84 (s, H-24), 1.02 (s, H-25), 0.83 (s, H-26), 0.79 (s, H-27), 0.95 (s, H-28), and 1.68 (s, H-30).

β**-Sitosterol (9)**

R_f_: 0.41 (*n*-Hexane-AcOEt, 8:2); ^13^C NMR (100 MHz, CDCl_3_) δ (ppm): 140.8 (C-5), 121.8 (C-6), 71.8 (C-3), 11.9 (C-18), 19.4 (C-19), 18.8 (C-21), 19.0 (C-26), 19.9 (C-27), and 12.0 (C-29); ^1^H NMR (400 MHz, CDCl_3_) δ (ppm): 5.36 (d, J = 5.8, ^1^H, H-6), 3.53 (m, ^1^H, H-3), 0.68 (s, 3H, H-18), 1.01 (s, 3H, H-19), 0.92 (d, J = 5.8 Hz, 3H, H-21), 0.80 (d, J=6.8 Hz, 3H, H-26), 0.82 (d, J=6.8 Hz 3H, H-27), and 0.84 (t, J=6.9 Hz 3H, H-29).

## 3. Results and discussion

Compound 1 was obtained as a white solid. The molecular formula was determined as C_22_H_26_O_10_ from the LC-Q-TOF-MS data (m/z 473.1412 [M+Na]^+^, calc. 473.1417). The UV spectrum of 1 showed absorption maxima at 324, 245, and 216 nm, typical of a caffeic acid derivative. The ^1^H NMR data were very similar to those of the caffeic acid moieties of chlorogenic acid methyl ester, but differed from known compounds with regard to the signals of the quinic acid moiety [25-26]. The ^1^H NMR spectrum of 1 (Table I) showed two pairs of doublets with coupling constants of 16.0 Hz indicative of trans olefinic protons found in hydroxy cinnamic acid. In the aromatic region, resonances for two ABX systems at δ 7.05 (d, J = 1.8 Hz, H-2′), 6.73 (d, J = 8.1 Hz, H-5′), and 6.92 ppm (d, J = 8.1 Hz, H-6′) were observed, which were assigned to the 1,3,4-trisubstituted phenyl units. From these observations, along with the analysis of the ^13^C and APT NMR data (Table 1), the presence of one caffeic acid moiety was inferred. The assignments were further supported by analysis of the COSY, HMBC, and HSQC spectrum of 1. The presence of the quinic acid moiety was indicated by ^1^H NMR resonances of three oxymethine protons at δ 5.39 ppm (m, ^1^H, H-3), 3.70 ppm (d, J = 8.3 Hz, H-4), and 4.14 (bs, ^1^H, H-5), together with two pairs of sp^3^ methylene protons at δ 2.15/2.05 ppm and 2.20/1.97 ppm for H-2a/2b and H-6a/6b, respectively. The latters are characteristics of a quinic acid unit, with regard to their multiplicity and coupling patterns. The assignments of the protons of the quinic acid nucleus were corroborated by analysis of the COSY and TOCSY spectra of 1. The attachment of caffeoyl moieties to the quinic acid part was deduced from the chemical sifts of H-3. The deshielded resonances of the oxymethine protons in the quinic acid nucleus at δ 5.39 ppm (H-3) implied acylation of the hydroxy group at these positions as reported before for other naturally occurring quinic acid derivatives [25-26]. The ^1^H NMR spectrum of compound 1 displayed a methoxy at δ 3.33 ppm (s, 3H) which was substitute to the quinic acid C-7 (169.01 ppm) that was shifted upfield. From these observations, the structure of 1 was initially thought to be that of the known compound 3-caffeoylquinic acid methyl ester (chlorogenic acid methyl ester). However, the ^1^H NMR spectrum of 1 showed slightly but distinctly different peak patterns of the (2-methylcyclopropyl) carbonyl unit at δ 1.78/1.60 (m, 2H, H-4′′), 1.05-0.88 (m, 3H, H-2′′, and H-3′′), and 1.02 (d, J = 6.8 Hz, 3H, H-5′′) and carbon peaks at δ 24.21 (CH_2_, C-4′′), 20.98 and 22.18 (2CH, C-2′′ and C-3′′), and 10.90 (CH_3_, C-5′′) which is substituted by ester linkage at C-1 position of quinic acid moiety of compound 1. All of the assigned NMR data of compound 1 is shown in table 1. Therefore compound 1 was elucidated as methyl 1-(2-methylcyclopropyl-1-carbonyloxy) chlorogenate.

**Figure 1:**
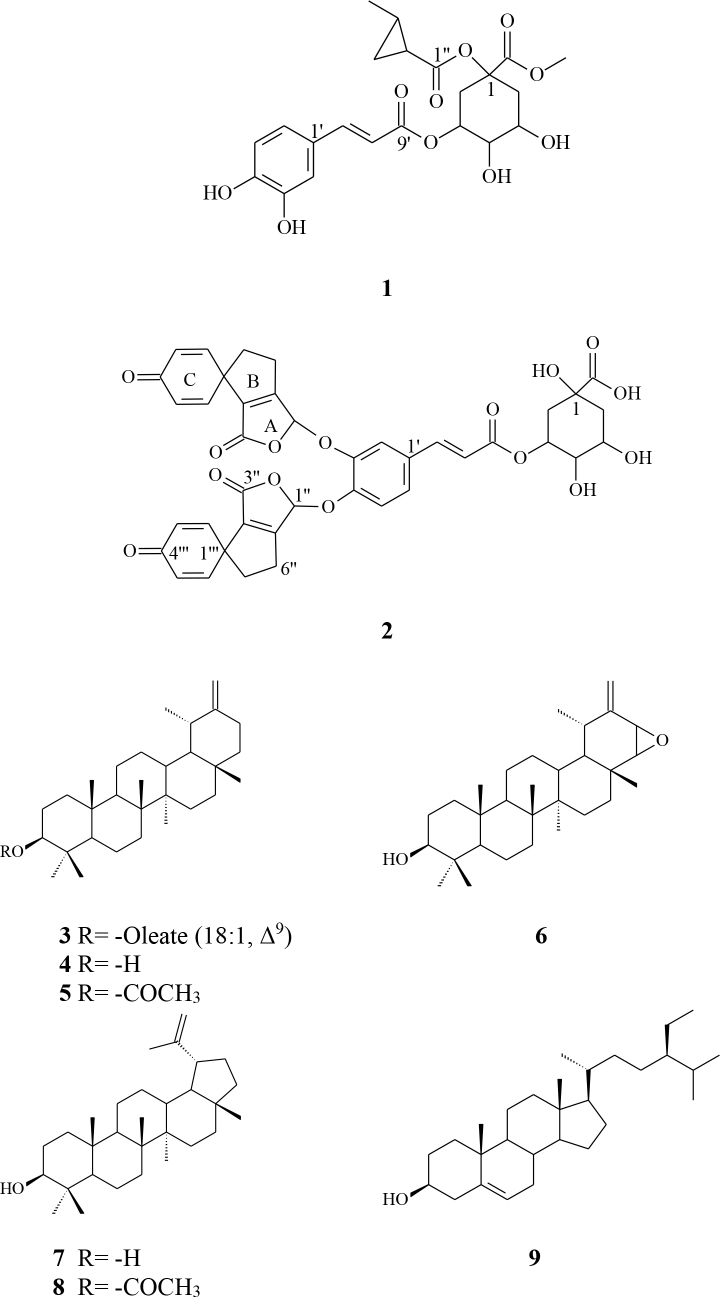


Compound 2 had a molecular formula of C40H34O15 with 24 unsaturated degrees as determined by NMR and LC-Q-TOF-MS data (m/z 753.6709 [M-H]^+^, calc. 753.6704). The FT-IR spectrum indicated the presence of an -OH group (3421 cm^-1^), carbonyl groups (1657 and 1597 cm^-1^), double bonds (1490 cm^-1^). The UV spectrum showed absorption maxima at 327, 243, and 218 nm which characteristic to caffeic acid moiety. The presence of a quinic acid moiety was characterized in the ^1^H NMR spectra of three oxymethine protons at δH 5.40 (m, H-3), 3.71 (d, J = 4 Hz, H-4), and 4.13 (bs, H-5), and the two pairs of sp^3^ methylene protons at δH 2.16/1.97 (2H, m, H-2a/2b) and 2.12/2.02 (2H, m, H-6a/6b) for H-2 and H-6 indicating the substitution at C-3 position. The presence of a mono substituted quinic acid moiety was suggested and then confirmed by the ^13^C NMR spectrum, δ C 76.38 (C, C-1), 37.62 (CH_2_, C-2), 71.71 (CH, C-3), 73.68 (CH, C-4), 71.18 (CH, C-5), and 39.29 (CH_2_, C-6). The ^1^H NMR spectrum indicated two protons of trans-configuration for double bonds, δ 7.59 (d, J = 16.0 Hz) and 6.30 (d, J = 16.0 Hz). In the aromatic region, resonances for three ABX systems, δH 7.05 (^1^H, d, J = 1.8 Hz), 6.77 (^1^H, d, J = 7.8 Hz), and 6.95 (^1^H, d, J = 7.8 Hz), were assigned to 1,3,4-trisubstituted phenyl unit. The part of the NMR data of compound 2 were very similar to those of the chlorogenic acid, but differed from known compounds with regard to the carbon signals of the caffeic acid moiety [25-26]. It was illuminated that the groups in the three-cyclic spiro structure were connected to the C-3′ and C-4 ′ positions of the caffeic acid part of compound 2. In the ^1^H NMR spectrum of compound 2, the acetal proton in ring A was at δ 7.00 ppm (s, 2H), the methylenic protons in ring B were at δ 3.57 (t, J= 7.3 Hz, 4H) and 2.25 (t, J = 7.3 Hz, 4H), and also the α,β-unsaturated protons in the ring C were at δ 7.11 (d, J = 12.0 Hz, 4H) and 6.25 (d, J = 12.0 Hz, 4H). The assignments of all moieties of compound 2 were further supported by analysis of the ^1^H-^1^H COSY and HMBC correlation (Figure 1). From these observations, along with the analysis of the ^13^C, APT, the COSY, HMBC, and HSQC NMR data of 2 (Table 2), the presence of two 3,4-bis[(3′,4-dioxo-1′,3′,5′,6′-tetrahydrospiro[cycclohexa-2,5-diene-1,4′-cyclopenta[c]furan]-1′-yl)] moiety was inferred. Thus, compound 2 was assigned as 3,4-bis[(3′,4-dioxo-1′,3′,5′,6′-tetrahydrospiro[cycclohexa-2,5-diene-1,4′-cyclopenta[c]-furan]-1′-yl)] chlorogenic acid, which was a new compound (2) and given the trivial name as scorzonerenone.

In the literature, chlorogenic acid and its derivatives have been isolated from various Scorzonera species and some of their biological activities have been investigated [27-34]. Petroleum ether, chloroform, ethyl acetate, and*n*-butanol fractions of ethanol extract of*Scorzonera hieraciifolia*Hayek have been tested in vitro antioxidant, antiinflammatory and antimicrobial activities, and ethyl acetate fraction has shown the highest amount of total phenolic content and antioxidant capacity; chlorogenic acid methyl ester derivatives were isolated from*S. hieraciifolia*[27]. Petroleum ether, CHCl_3_, ethyl acetate, and*n*-BuOH fractions of an EtOH extract obtained from subaerial parts of*Scorzonera pygmaea*had tested in vitro antioxidant, antiinflammatory, and antimicrobial activities, which yielded chlorogenic acid and methyl esters from*S. pygmaea*[28]. Five new quinic acid derivatives and two known 3-O-feruloylquinic acids have been isolated from the roots of*Scorzonera divaricata*Turcz. Quinic acid derivative exhibited strong antioxidant activity, with IC50 values of 3.95, 5.87, and 7.45 μg/mL against ABTS+ and 11.7, 13.6, and 50.1 μg/mL against DPPH [29]. Quinic acid derivatives from aerial parts of*S. radiata*were isolated and their antioxidant activities have been evaluated by the DPPH assay [30]. The aerial parts of*Scorzonera aristata*Ramond ex DC., have given chlorogenic acid derivatives and the triterpenes 3*α*-hydroxyolean-5-ene, lupeol, and magnificol [31]. Antiinflammatory activity of*Scorzonera*extracts in vivo*S. latifolia*,*Scorzonera cana*var.*jacquiniana*,*Scorzonera tomentosa*,*Scorzonera mollis*ssp.*szowitsii*,*Scorzonera eriophora*,*Scorzonera incisa*,*Scorzonera cinerea*, and*Scorzonera parviflora*has been evaluated for their inhibitory activities of TNF-*α*and IL-1*β*production, and NF-κB nuclear translocation in THP-1 macrophages and HPLC analysis revealed that the chlorogenic acid was present in all tested extracts [32]. Isolation of caffeic acid derivatives was mentioned from the subaerial parts of*S. latifolia*[33]. Chlorogenic acid derivatives and chlorogenic acid methyl ester were reported from*Scorzonera veratrifolia*Fenzl [34].

In this research, the structure of one new and six known triterpenoids from the aerial parts of*S. aucheriana*were isolated by CC. The new triterpenoids were taraxasterol oleate (3) and knowns as: taraxasterol (4), taraxasterol acetate (5), ptiloepoxide (6), lupeol (7), lupeol acetate (8), and β-sitosterol (9). The structures of these compounds were elucidated by ^1^H, ^13^C, APT, and COSY spectra, and their data were compared with the reported data obtained from the literature. Triterpene compounds containing long chain fatty acids were characterized from natural sources. Of the peaks observed in the proton and carbon NMR spectrum of compound 3, the triterpene ring was detected from the peaks in the proton and carbon NMR spectra (80.6 (C-3), 154.6 (C-20), 107.2 (C-30), and seven methyl peaks at δ 27.9 (C-23), 16.3 (C-24), 16.6 (C-25), 15.8 (C-26), 14.7 (C-27), 19.5 (C-28), 25.5 (C-29) and 4.42 (m, ^1^H, H-3), 0.78 (s, 3H, H-23), 0.77 (s, 3H, H-24), 0.81 (s, 3H, H-25), 0.81 (s, 3H, H-26), 0.83 (s, 3H, H-27), 0.86 (s, 3H, H-28), 0.89 (d, J = 6.8 Hz, 3H, H-29), 4.44 (brs, H-30a), 4.40 (brs, H-30b)), where the triterpene ring was taraxasterol. ^1^H NMR spectra for the aglycone portion of compound 3 revealed peaks at δ 5.32 (m, 2H, H-9, H-10), 2.33 (t, J = 6.8 Hz, 2H, H-2), 1.20 (t, J = 6.8 Hz, 3H, H-18) and the carbonyl peak at δ 171.00 ppm, olefinic carbon peaks at δ 130.1 (=CH, C-9), 129.6 (=CH, C-10), and terminal methyl peak at δ 14.1 ppm (-CH_3_, C-18) in the ^13^C NMR spectrum suggested that glycone part of compound 3 was oleic acid. Thus, compound 3 was characterized as taraxasterol olaeate (urs-20(30)-en-3-ol, 3β-[(9Z)-9-octadecenoate]) and was not found in the literature. However, the similar compounds (urs-20(30)-en-3-ol, octadecanoate, urs-20(30)-en-3-ol, eicosanoate, urs-20(30)-en-3-ol, 3-hexadecanoate, and urs-20(30)-en-3-ol, 3-dodecanoate) were mentioned in the literature [35-38].

The ^1^H NMR spectrum of compound 4 revealed a multiple at δ 3.21 (d, J = 6.3, 13.2) for the H-3 and methylene protons at δ 4.60 (brs, H-30a), and 4.62 (brs, H-30b) ppm as two broad singlets. The same spectrum showed signals for methyls at δ 0.85 (s, 3H, H-23), 0.87 (s, 3H, H-24), 1.01 (s, 3H, H-25), 0.93 (s, 3H, H-26), 0.85 (s, 3H, H-27), 0.77 (s, 3H, H-28), and 1.02 (d, J = 6.8 Hz, 3H, H-29). The ^13^C NMR spectrum showed 30 signals. The peaks at δ 154.7 (C-20) and 107.2 (C-30) are related to the olefinic carbons. The methine signal at δ 79.0 ppm was for C-3. The peaks at δ 28.0 (C-23), 15.4 (C-24), 16.3 (C-25), 15.9 (C-26), 15.4 (C-27), 19.5 (C-28), and 25.6 (C-29) ppm were related to the methyl carbons. Compound 4 was characterized as taraxasterol [39]. Compound 5 showed similar NMR data as compound 4. However, in the ^1^H NMR spectra of compound 5, the peaks at δ 4.45 ppm (m, ^1^H) for H-3 and, the singlet peak at δ 2.05 ppm (s, 3H) for COCH_3_, and the carbonyl peak at δ 171.00 ppm in the ^13^C NMR spectrum suggested that compound 5 was taraxasterol acetate [40].

The ^1^H NMR spectrum of compound 6 displayed a dd at δ 3.21 (J = 11.3, 5.0 Hz, ^1^H) for the H-3. This is deshielded proton as a result of -OH substitution. ^1^H NMR spectrum of compound 6 displayed methylene protons at δ 5.06 (s, H-30a) and 4.87 (s, H-30b) ppm as two singlets and epoxide protons at δ 3.47 (d, J = 4.6 Hz, ^1^H, H-21), and at δ 2.91 ppm (d, J = 4.6 Hz, ^1^H, H-22). The same spectrum showed signals for Me-23 at δ 0.97 (s), Me-24 at δ 0.77 (s), Me-25 at δ 0.84 (s), Me-26 at δ 1.02 (s), Me-27 at δ 0.95 (s), Me-28 at δ 0.81 (s), and Me-29 at δ 1.05 (d, J = 6.8 Hz). The ^13^C NMR spectral data showed 30 signals. The peaks at δ 151.3 (C-20) and 112.0 (C-30) ppm are related to the olefinic carbons. The methines signals at δ 78.9 for C-3 and at δ 56.1 (C-21), 64.0 (C-22) ppm related to the epoxide carbons. Compound 6 was identified to be ptiloepoxide [41].

The ^1^H NMR spectrum of compound 7 gave a peak at δ 3.20 (dd, 10.4, 4.9 Hz,) for the H-3 and methylene protons at δ 4.69 (bs, J = 0.3 Hz, ^1^H, H-21a), and δ 4.57 (bs, J = 0.3 Hz, ^1^H, H-21b) ppm. The same spectrum showed seven signals for methyl peaks at δ 0.76 (s, H-20), 0.83 (s, H-24), 1.03 (s, H-25), 0.97 (s, H-26), 0.79 (s, H-27), 1.68 (s, H-28), and 1.68 (s, H-29) ppm. The ^13^C NMR spectrum showed 29 signals. The peaks at δ 151.0 (C-19) and 109.3 (C-21) are related to the olefinic carbons. The methine signal at δ 79.0 ppm was for C-3. The peaks at δ 28.0 (C-20), 15.4 (C-24), 16.1 (C-25), 16.0 (C-26), 14.6 (C-27), 18.0 (C-28), and 19.3 (C-29) ppm were related to the methyl carbons. Compound 7 was elucidated as lupeol [42]. Compound 8 revealed the similar NMR data as compound 7. However, in the ^1^H NMR spectra of compound 8, the peaks at δ 4.48 ppm (m, ^1^H) for H-3 and, the singlet peak at δ 2.05 ppm (s, 3H) for COCH_3_, and the carbonyl peak at δ 171.09 ppm in the ^13^C NMR spectrum supported that compound 8 was lupeol acetate [43].

The ^1^H NMR spectrum of compound 9 showed a multiple peak at δ 3.53 (m, ^1^H) for the H-3 and CH proton at δ 5.36 (d, J = 5.8, ^1^H, H-6) ppm. The same spectrum showed signals for methyl at δ 0.68 (s, 3H, H-18), 1.01 (s, 3H, H-19), 0.92 (d, J = 6.6 Hz, 3H, H-21), 0.80 (d, J = 6.8 Hz, 3H, H-26), 0.82 (d, J = 6.8 Hz, 3H, H-27), 0.84 (t, J = 6.9 Hz, 3H, H-29). The ^13^C NMR spectral data showed 29 signals. The peaks at δ 140.8 (C-5), 121.8 (C-6), are related to the olefinic carbons. The methine signal at δ 71.8 ppm was for C-3. The peaks at δ 11.9 (C-18), 19.4 (C-19), 18.8 (C-21), 19.0 (C-26), 19.9 (C-27), and 12.0 (C-29) ppm were related to the methyl carbons. Compound 9 was identified as β-sitosterol [44].

Triterpenes are common natural compounds for the various Scorzonera species, which were mentioned in the liteature [45-53]. Isolation of three tirucallane triterpenes were mentioned from roots of*S. divaricata*and exhibited significant cytotoxic activities in vitro against four human cancer cell lines (HL60, HeLa, HepG2, and SMMC-7721) [45].*n*-Hexane extract of*S. latifolia*roots has given 3*β*-hydroxyfern-7-en-6-one-acetate, urs-12-en-11-one-3-acetyl, 3*β*-hydroxy-fern-8-en-7-one-acetate, olean-12-en-11-one-3-acetyl, and leucodin [8]. D:B-friedoolean-type triterpene has been isolated from the roots of*S. austriaca*and in vitro cytotoxicities of triterpenes had tested to cancer cells of human promyelocytic leukemia (HL-60) and human hepatoma (BEL-7404) [46]. Isolation of triterpenes (lupeol, betulin, betulinic acid, lupeo acetate, 23Z-3*β*-acetoxyeupha-7,23-diene-25-ol, dammaradienyl acetate, pulcherryl acetate, taraxasterol, taraxasteryl acetate,*ψ-*taraxasterol,*ψ*-taraxasteryl acetate,*α*-amyrin acetate, dammar-24-ene-3,20-diol,3*β*-tetradecanoate, butyrospermyl acetate and multiflorenyl acetate) were reported from*S. mongolica*. Lupeol, betulin, betulinic acid, lupeo acetate, and 23Z-3*β*-acetoxyeupha-7,23-diene-25-ol had demonstrated significant inhibitory effects on A-549 cancer cells at the concentration of 50 mg/L-1 [47]. Two erythrodiol triterpene fatty esters (3*β*-dodecanoyl erythrodiol and 3*β*-tetradecanoyl erythrodiol) were characterized from*S. mongolica*and their antitumor effects in vitro has been evaluated with MTT and SRB assays, but showed moderate cytotoxicity on A-549 cell line [48].*β*-Sitosterol, cholesterol, and other triterpenes have been isolated from*S. mongolica*[49]. From the roots of*Scorzonera undulata*ssp.*deliciosa*(Guss.) Maire*β*-amyrin acetate, stigmasterol, and*β*-sitosterol were reported [50]. Two new triterpene fatty esters (3*β*-tetradecanoyloxy-28-hydroxylolean-18-ene and 3*β*-dodecanoyl-28-hydroxylolean-18-ene) were characterized by*S. mongolica*[51]. Isolation of*β*-amyrin acetate, methyl oleanate, methyl ursolate, stigmasterol, and*β*-sitosterol was given from*S. undulata*ssp.*deliciosa*and the methanol extract had examined for in vitro antioxidant properties using DPPH test [52].*n*-Hexane extract of the*S. latifolia*had yielded*α*-amyrin, lupeol, lupeol acetate, taraxasteryl acetate, 3*β*-hydroxy-fern-7-en-6-one acetate, 3-acetyl urs-12-en-11-one, 3*β*-hydroxy-fern-8-en-7-one acetate, and 3-acetyl olean-12-en-11-one [53].

## 4. Conclusion

Hexane and ethyl acetate fraction of a crude methanol extract of the*S. aucheriana*afforded nine natural compounds. Two chlorogenic acid derivatives were isolated as new compounds ( 1 and 2). Seven triterpenoids and derivatives were also isolated, and taraxasterol oleate (3) was found to be a new compound along with six known (4-9) ones. All compounds (1-9) were isolated and identified for the first time from this species.
